# BET bromodomain inhibitors attenuate transcription of a subset of IL-1-induced NF-κB targets that promote inflammation in β-cells

**DOI:** 10.1016/j.jbc.2025.110358

**Published:** 2025-06-10

**Authors:** Joshua A. Nord, Savannah J. Makowski, Paul F.W. Sidlowski, Karina L. Bursch, John A. Corbett, Brian C. Smith

**Affiliations:** 1 Department of Biochemistry, Medical College of Wisconsin, Milwaukee, Wisconsin, USA; 2 Structural Genomics Unit, Linda T. and John A. Mellowes Center for Genomic Sciences and Precision Medicine, Medical College of Wisconsin, Milwaukee, Wisconsin, USA; 3 Program in Chemical Biology, Medical College of Wisconsin, Milwaukee, Wisconsin, USA

**Keywords:** β-cell, inflammation, BET bromodomains, Bromodomain-containing protein 4 (BRD4;, NF-κB transcription factor, diabetes, epigenetics, transcription, small molecule, gene regulation

## Abstract

Cytokine-stimulated transcription of NF-**κ**B target genes is linked to the development of multiple inflammatory and autoimmune diseases. Inhibitors of bromodomain and extraterminal domain (BET) epigenetic reader proteins attenuate inflammatory gene transcription and delay the onset of several inflammatory diseases, including autoimmune diabetes. Our previous studies showed that BET bromodomain inhibitors disrupt the interaction between BET family member BRD4 and NF-κB transcription factor p65 in **β**-cells, thus attenuating cytokine-stimulated NF-**κ**B-dependent gene and functional changes. However, the role of NF-κB in developing inflammatory disease is controversial, as NF-**κ**B inhibition can promote disease progression in some contexts. NF-**κ**B target genes play both physiological and pathophysiological roles in regulating the cellular response to cytokines. Here, using cytokine-stimulated pancreatic **β**-cells as an inflammatory disease model, we show that NF-**κ**B-dependent gene products that participate in inflammation are sensitive to BET bromodomain inhibition. In contrast, gene products that maintain cellular homeostasis or protect **β**-cells from stressors are largely insensitive to BET bromodomain inhibition. These studies define a novel and selective role for BET bromodomain-containing proteins in regulating inflammatory gene activation.

Inflammatory diseases are generally accompanied by alterations in histone modification patterns and epigenetic protein activity that can trigger amplified and/or sustained transcriptional activation of pro-inflammatory genes (1, 2). The bromodomain and extraterminal domain (BET) family of epigenetic regulatory proteins (BRD2, BRD3, BRD4, and BRDT) regulate inflammatory gene transcription [reviewed in (3)]. Each BET family member contains two bromodomains that bind acetylated lysine residues on histones and other nuclear proteins to modify transcription (4, 5). The BET family exerts broad anti-inflammatory effects across many disease models, including arthritis (6), fibrosis (7, 8), coronary artery disease (9), cardiovascular disease (10), and diabetes (11, 12). In autoimmune diabetes (11, 12), pan-BET bromodomain inhibitors [I-BET151 (13) and I-BET762 (14)] attenuate the onset of insulitis and progression to diabetes in the non-obese diabetic (NOD) mouse (11, 12, 15).

The anti-inflammatory effects of BET bromodomain inhibitors are attributed mainly to the attenuation of nuclear factor kappa B (NF-κB) activity (3, 16, 17, 18). As a “master regulator” of inflammation, NF-κB is activated by signaling cascades that stimulate phosphorylation and degradation of the inhibitor protein IκB. This allows NF-κB to translocate to the nucleus and induce the transcription of pro-inflammatory gene targets (19). Aberrant activation of NF-κB signaling is strongly linked to autoimmune and inflammation-related disease development (20, 21, 22). For example, expression of a constitutively active NF-κB mutant selectively in pancreatic β-cells results in the spontaneous development of immune-mediated diabetes in mice (23). Mechanistically, BRD4 can interact with acetylated p65, the primary subunit of NF-κB, to activate the transcription of pro-inflammatory NF-κB targets (17, 18, 24). We recently showed that BET bromodomain inhibition disrupts the interaction of BRD4 with p65 and attenuates cytokine-induced NF-κB transcriptional activity in β-cells (25). Overall, these findings suggest that attenuating inflammatory gene expression through BET bromodomain inhibition is a consequence of disrupting the interaction between BET proteins and NF-κB.

Paradoxically, pharmacologically or genetically inhibiting NF-κB increases inflammation and cell toxicity in some contexts [reviewed in (26)]. Inhibiting NF-κB *via* expressing a non-degradable form of IκBα in β-cells accelerates the development of diabetes in NOD mice (27). These findings indicate that the genes regulated by NF-κB are diverse and that not all NF-κB targets are damaging. Indeed, NF-κB also activates the transcription of genes required for maintaining cellular homeostasis, cell survival, proper immune function, and inhibiting inflammation (26). The protective actions of BET bromodomain inhibitors compared to the disparate effects of direct inhibition of NF-κB on diabetes development suggest that BET bromodomain inhibitors may selectively modify NF-κB target gene transcription. Indeed, we and others have identified several NF-κB-regulated target genes insensitive to BET bromodomain inhibition (17, 18, 25).

In this study, we tested the hypothesis that BET bromodomain inhibitors modify the expression of only a subset of cytokine-induced NF-κB gene targets in β-cells. This was performed in a β-cell-like insulinoma cell line (INS 832/13) treated with interleukin-1 (IL-1) to activate NF-κB (28). While the actions of IL-1 have historically been viewed as damaging to β-cells (29, 30, 31), we recently showed that IL-1 also stimulates the transcription of genes whose products function in the defense against environmental stressors, including viral infection (32, 33, 34). Using RNA-sequencing (RNA-seq), gene set enrichment analysis (GSEA) (35, 36), and Database for Annotation, Visualization and Integrated Discovery (DAVID) (37, 38), we identified heterogeneity in the categories of genes sensitive to BET bromodomain inhibition in IL-1-treated β-cells. We show that BET bromodomain inhibitors attenuate the expression of NF-κB-dependent genes involved in inflammation. In contrast, IL-1-induced gene products that regulate cellular homeostasis and protect from environmental stressors are largely insensitive to BET bromodomain inhibition. These studies begin to define the mechanisms of regulation and classes of NF-κB-dependent genes controlled by BET proteins in inflammatory diseases.

## Results

### IL-1 induces rapid gene expression changes in **β**-cells

**Figure 1:**
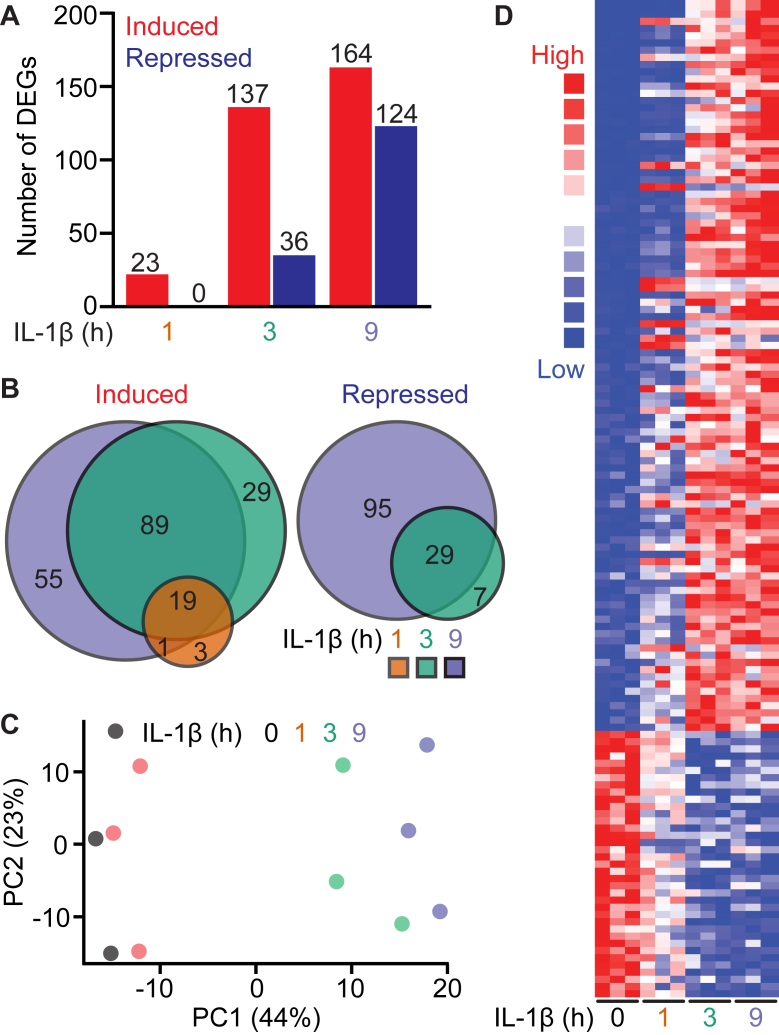
**IL-1 modulates gene expression in INS 832/13 cells**. *A*, number of genes induced or repressed (fold-change > 1.5; adjusted *p* < 0.05) by 5 U/ml IL-1β in INS 832/13 cells at indicated treatment time points. *B,* Venn diagrams displaying overlapping genes within induced or repressed categories at indicated IL-1β exposures. *C*, PCA plot displaying variability across biological replicates and treatment timepoint. *D*, Heatmap of differentially expressed genes across IL-1β treatment time points.

Most transcriptome analyses of cytokine-treated islets or insulinoma cells have been performed following 24- or 48-h cytokine treatments, well after impairments in β-cell function are observed (39, 40, 41). These prolonged cytokine treatments miss early gene expression changes, as evidenced by our recent single-cell RNA-sequencing (scRNA-seq) studies in mouse and human islets following 6-h cytokine treatments (32, 33). To elucidate early changes in gene expression more directly related to BET bromodomain function, INS 832/13 β-like-cells were exposed to 5 U/ml IL-1 for 1, 3, and 9 h, then subjected to bulk RNA-seq. Differences in the levels of RNA transcript between IL-1 treated and untreated INS 832/13 cells of >1.5-fold with an adjusted *p*-value < 0.05 were used to identify differentially expressed genes (DEG). We found that IL-1 stimulates rapid changes in gene expression, with 23 genes displaying increased expression after 1 h of exposure (Fig. 1*A*). Following a 3-h exposure, the expression of 137 genes was increased, and following a 9-h exposure, IL-1 increased the expression of 164 genes (Fig. 1*A*). Temporally, nearly 80% of the genes with increased expression after 1 h remained elevated after 9 h of IL-1 exposure. We did not observe decreases in gene expression following a 1-h exposure; however, following 3- and 9-h exposure, IL-1 decreased the expression of 36 and 124 genes, respectively (Fig. 1*A*). Similarly, more than 80% of the genes with decreased expression after 3 h remain repressed after 9 h of IL-1 exposure (Fig. 1*B*). Principal component analysis (PCA) (Fig. 1*C*) and the DEG heatmap (Fig. 1*D*) confirmed the substantial alterations in gene expression induced by IL-1 as compared to untreated INS 832/13, with similarities observed in the expression of genes in INS 832/13 cells treated for 3 and 9 h with IL-1.

### IL-1 induces inflammatory responses and a loss of functional identity in **β**-cells

**Figure 2:**
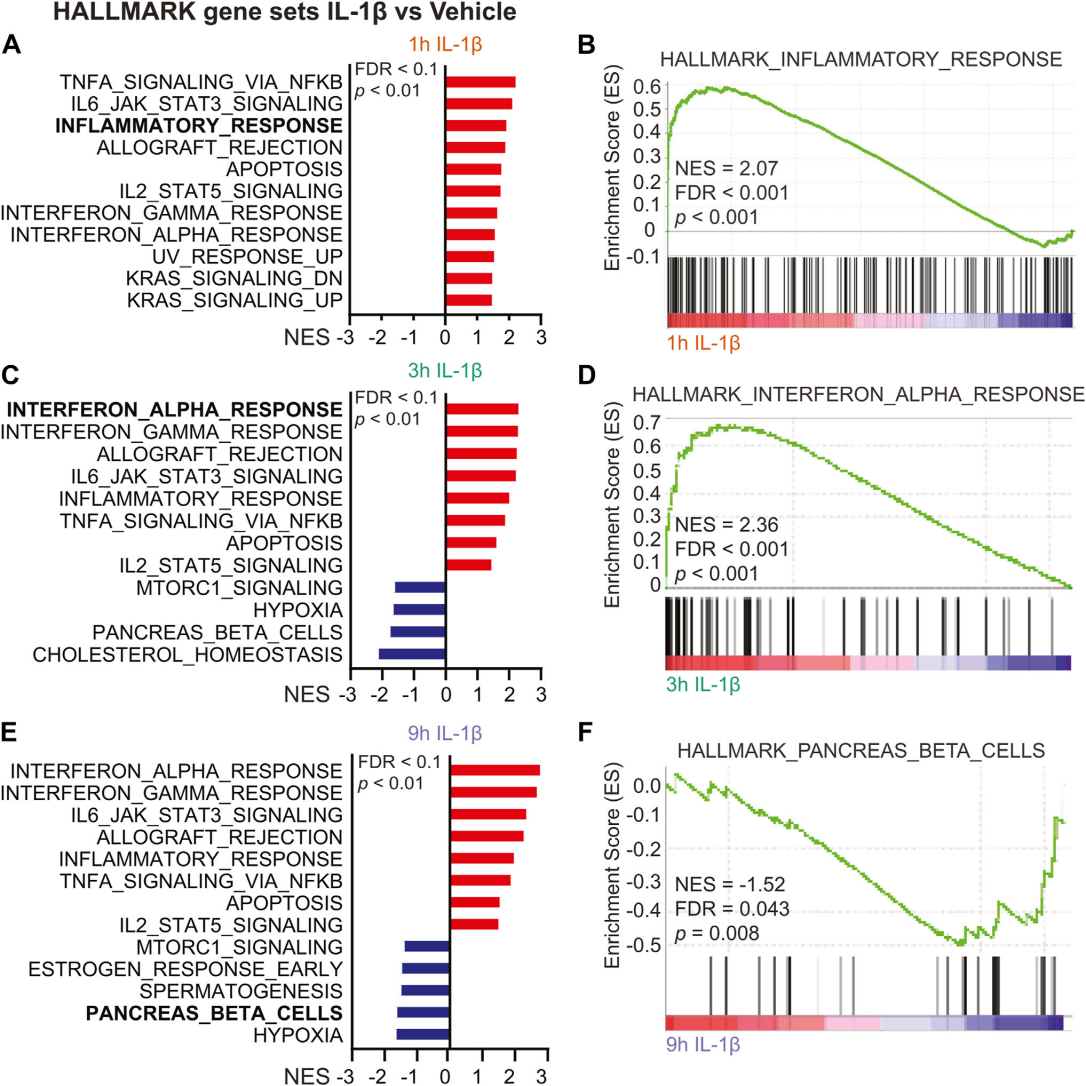
**IL-1 induces the expression of inflammatory gene sets and represses pancreatic β-cell genes in INS 832/13 cells**. *A/C/E*, HALLMARK pathway normalized enrichment scores (NES) for enriched or repressed gene sets (FDR < 0.1, *p* < 0.01) following indicated exposure to 5 U/ml IL-1β. *B/D/F*, HALLMARK GSEA plots for select gene sets identified in (*A/C/E*).

Unbiased Gene Set Enrichment Analysis (GSEA) with Hallmark annotations were used to analyze and identify pathways in which gene expression is significantly modified in response to IL-1 (*p* < 0.01; false discovery rate [FDR] < 0.1). Predictably, gene sets associated with the induction and maintenance of inflammation (*e.g.* inflammatory response, TNFα signaling, interferon responses, apoptosis) were induced by IL-1 (Fig. 2, *A*–*E*). Many of these processes were induced as early as 1 h of IL-1 exposure (Fig. 2, *A*/B) and remained elevated following 3- and 9-h exposures (Fig. 2, *C*–*E*). The primary class of genes repressed by IL-1 was pancreas β-cell genes, and this was observed following 3 and 9 h of IL-1 exposure (Fig. 2, *C*/E/F). Functional annotation clustering analysis by DAVID was also performed on the RNA-seq data set (identified in Fig. 1*A*) and largely corroborated the GSEA results, as the top gene ontology (GO) terms identified were similar to those identified by GSEA (*e.g.* inflammatory response, cellular response to TNF, cellular response to type II interferon) (Fig. S1). GSEA did not identify additional terms related to inflammatory pathways, including immune response, defense response, and other chemokine-related responses (Fig. S1). These findings are consistent with our previous studies and the work of others that identified induction of inflammatory genes and loss of identity genes in β-cells treated with IL-1 (32, 33, 34, 42).

### Selective regulation of IL-1 stimulated genes in **β**-cells by BET inhibitors

**Figure 3:**
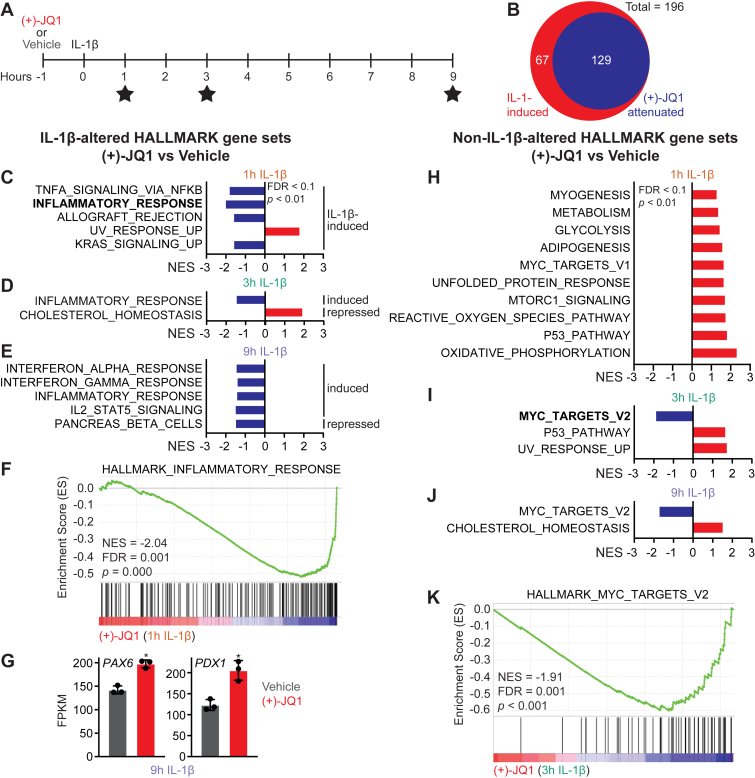
**BET bromodomain inhibitors attenuate IL-1-induced transcription of inflammatory gene sets**. *A*, Schematic representation of treatment schedule with stars indicating harvest time points. *B*, Venn diagram indicating the proportion of IL-1β-induced genes significantly attenuated (fold-change > 1.5; adjusted *p* < 0.05) by 0.5 μM (+)-JQ1. *C–E*, HALLMARK pathway normalized enrichment scores (NES) for 0.5 μM (+)-JQ1 *versus* vehicle for IL-1β altered gene sets (FDR < 0.1, *p* < 0.01). *F***,** HALLMARK GSEA plot of inflammatory response genes for 0.5 μM (+)-JQ1 *versus* vehicle control at 1 h IL-1β exposure. *G***,** plotted FPKM expression values from RNA-seq analysis for PAX6 and PDX1 comparing 0.5 μM (+)-JQ1 to vehicle controls at 9 h IL-1β exposure. Three biological replicates are denoted as individual points with significance calculated using an unpaired Student's *t* test, ∗ < 0.05, and error bars represent SD. *H–J*, HALLMARK pathway normalized enrichment scores (NES) for 0.5 μM (+)-JQ1 *versus* vehicle in non-IL-1β altered gene sets at indicated IL-1β exposures. *K***,** HALLMARK GSEA plot for MYC target genes for 0.5 μM (+)-JQ1 *versus* vehicle control at 3 h of IL-1β exposure.

Previously, we showed that BET bromodomain inhibitors attenuate cytokine-stimulated transcription of inflammatory genes *NOS2* and *PTGS2* in INS 832/13 cells and primary β-cells (25). To globally probe the impacts of BET bromodomain inhibition on inflammatory gene transcription in β-cells, INS 832/13 cells were pretreated with the pan-BET bromodomain inhibitor (+)-JQ1 (43) for 1 h, IL-1 was added, and the cells were cultured for 1, 3, or 9 additional hours. The cells were harvested and subjected to bulk RNA sequencing (Fig. 3*A*). Approximately two-thirds of 196 differentially expressed genes induced by IL-1 were sensitive to (+)-JQ1 inhibition (>1.5-fold attenuation; adjusted *p*-value < 0.05) (Fig. 3*B*). GSEA analysis revealed significant overlap in the Hallmark gene sets induced by IL-1 (Fig. 2, *A*–*E*) and the Hallmark gene sets sensitive to (+)-JQ1 at each time point examined (Fig. 3, *C*–*E*, blue bars; Fig. 3*F*). This includes Hallmark gene sets involved in inflammation and inflammatory mediator signaling. As multiple cytokines stimulate transcription of similar gene sets, among the gene sets induced by IL-1 and attenuated by (+)-JQ1 were signatures associated with exposure to other cytokines, including TNFα signaling and interferon responses (Fig. 3, *C*/E).

In contrast, the only category of genes with increased expression in response to (+)-JQ1 were UV response genes following 1-h IL-1 exposure (Fig. 3*C*, red bar), while (+)-JQ1 reversed the repression by IL-1 of genes involved in cholesterol homeostasis at 3 h (Fig. 3*D*, red bar). Except for the Hallmark gene set pancreatic β-cell genes, (+)-JQ1 broadly reversed many gene sets modulated by 9-h IL-1 exposure (Fig. 3*E*). This finding contrasts with evidence that BET bromodomain inhibition increases the expression of several β-cell identity genes (41). Although the pancreatic β-cell gene set as a whole is attenuated by (+)-JQ1, we confirmed that the expression of β-cell identity genes *PAX6* and *PDX1*, the master regulator of β-cell maturation, function, and identity (44) is increased by (+)-JQ1 (Fig. 3*G*).

Several Hallmark gene sets were not altered by IL-1 alone (Fig. 2) but increased in the presence of IL-1 and (+)-JQ1. These included Hallmark gene sets associated with metabolic processes, including metabolism, glycolysis, oxidative phosphorylation, unfolded protein response, reactive oxygen pathways, and cholesterol homeostasis (Fig. 3, *H*–*J*). Genes associated with adipogenesis were also induced by (+)-JQ1 (Fig. 3*H*). Consistent with the known attenuation of *MYC* expression by BET bromodomain inhibition (45, 46), (+)-JQ1 repressed MYC target genes and stimulated p53 target genes (Fig. 3, *H*–*K*). These findings are consistent with previous reports that BET proteins are co-activators of inflammatory gene transcription and co-repressors of select metabolic and adipocyte functional genes known to be PPARγ-regulated (47).

### IL-1 induces the transcriptional activation of NF-**κ**B-regulated target genes

**Figure 4:**
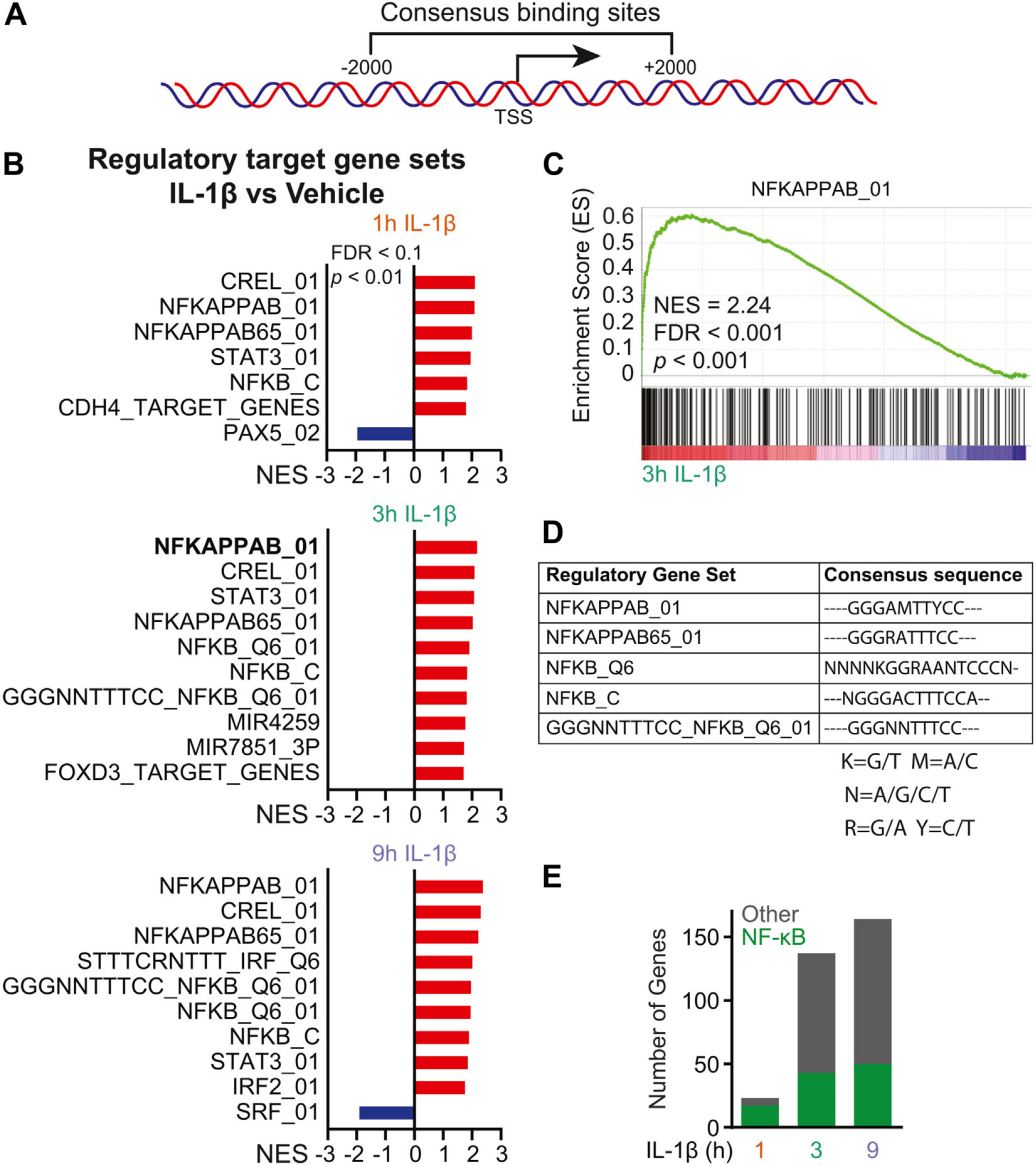
**IL-1 induces transcription of NF-κB target genes in INS 832/13 cells**. *A*, schematic representation of the regulatory region. *B*, regulatory target normalized enrichment scores (NES) for enriched or repressed regulatory target gene sets following indicated exposure to 5 U/ml IL-1β (FDR < 0.1, *p* < 0.01). *C*, regulatory target GSEA plot for NFKAPPAB_01 target genes for 0.5 μM (+)-JQ1 *versus* vehicle control at 3 h of IL-1β exposure (FDR < 0.1, *p* < 0.01). *D*, table of identified NF-κB regulatory target gene sets and their consensus sequences. *E*, Stacked bar graph displaying the total number of IL-1β-induced genes and those containing at least one NF-κB consensus site within their regulatory region (*green*).

We previously found that the BET protein BRD4 interacts with the p65 subunit of NF-κB to activate the transcription of multiple inflammatory genes in β-cells, including *NOS2* (25). GSEA for regulatory target gene sets was used to probe for potential BET protein binding partners that may function as co-activators of cytokine-induced inflammatory gene transcription in β-cells. This computational approach uses known or predicted consensus elements for transcription factors and/or microRNAs that share *cis*-regulatory elements with the differentially expressed genes (Fig. 4*A*). Consistent with known BRD4 interactions with the NF-κB subunit p65 (25), NF-κB was the primary transcription factor binding element in genes induced by IL-1 (Fig. 4*B*, red bars; *C*). Five of the most prominent enriched gene sets contain conserved NF-κB consensus sequences, encompassing the largest group of IL-1-induced genes (Fig. 4, *D* and *E*). In agreement with previous studies identifying the STAT transcription factor family as regulators of multiple inflammatory genes [reviewed in (48)], STAT3 was the second most enriched consensus element amongst IL-1-induced genes (Fig. 4*B*).

**Figure 5:**
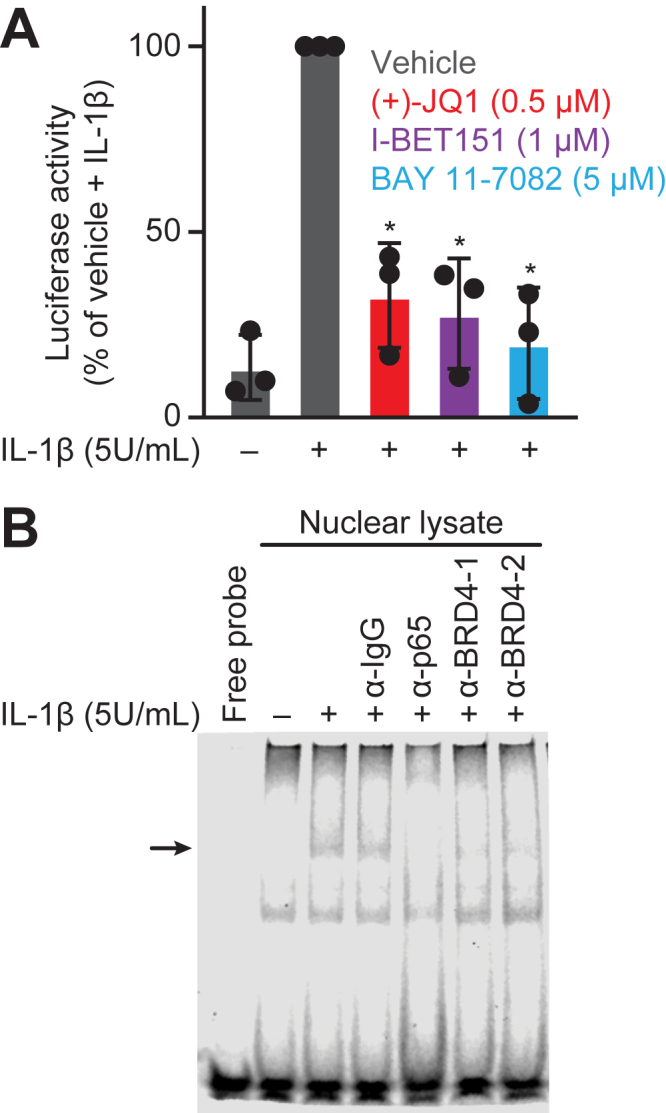
**Assessment of BET inhibition on NF-κB activity**. *A*, NF-κB reporter assay in INS 832/13 cells transfected with a 3x-KB-L luciferase plasmid (Addgene #26699), treated with 0.5 μM (+)-JQ1, 1 μM I-BET151, 5 μM BAY 11-7082, or vehicle control for 1 h, followed by the addition of 5 U/ml IL-1β for 5 h. Three biological replicates are denoted as individual points with significance calculated using an unpaired Student's *t* test, ∗ < 0.05, and error bars represent SD. *B*, gel shift analysis of nuclear extracts isolated from INS 832/13 cells exposed to 5 U/ml IL-1β for 30 min using a fluorescently-labeled NF-κB probe and antibodies specific for p65 or BRD4. Bands represent free probe or NF-κB shift (*black arrow*). A representative gel is shown, n = 2.

Having previously demonstrated that BET bromodomain inhibitors attenuated IL-1-induced NF-κB transcriptional activity of *NOS2* and previously shown that the BRD4 tandem bromodomains interact with p65 in cells (25), we next sought to link the effects of BET bromodomain inhibition directly to NF-κB signaling. First, a reporter plasmid containing three NF-κB binding elements upstream of a minimal promoter and luciferase (3x-KB) was used. We confirmed that pan-BET bromodomain inhibitors (+)-JQ1 and I-BET151 (13) attenuate NF-κB reporter activity (Fig. 5*A*). As a positive control, we also show that the NF-κB inhibitor BAY 11-7082, which inhibits IKK activity (49), attenuates NF-κB reporter activity in a manner similar to the BET bromodomain inhibitors (Fig. 5*A*). Next, we used gel shift analyses to determine if BRD4 and p65 can co-localize at the NF-κB consensus sequence. Nuclear extracts were obtained from control and IL-1-stimulated INS 832/13 cells and incubated with a fluorescently-labeled DNA probe matching the consensus sequence for NF-κB. A 30-min exposure to IL-1 induced nuclear localization of NF-κB subunits as evidenced by reduced mobility of the DNA probe. Both p65 and BRD4 are present in this NF-κB complex, as evidenced by diminished intensity of the shifted band when incubated with antibodies directed against p65 or BRD4 (Fig. 5*B*).

### BET bromodomain inhibitors differentially attenuate NF-**κ**B target genes

**Figure 6:**
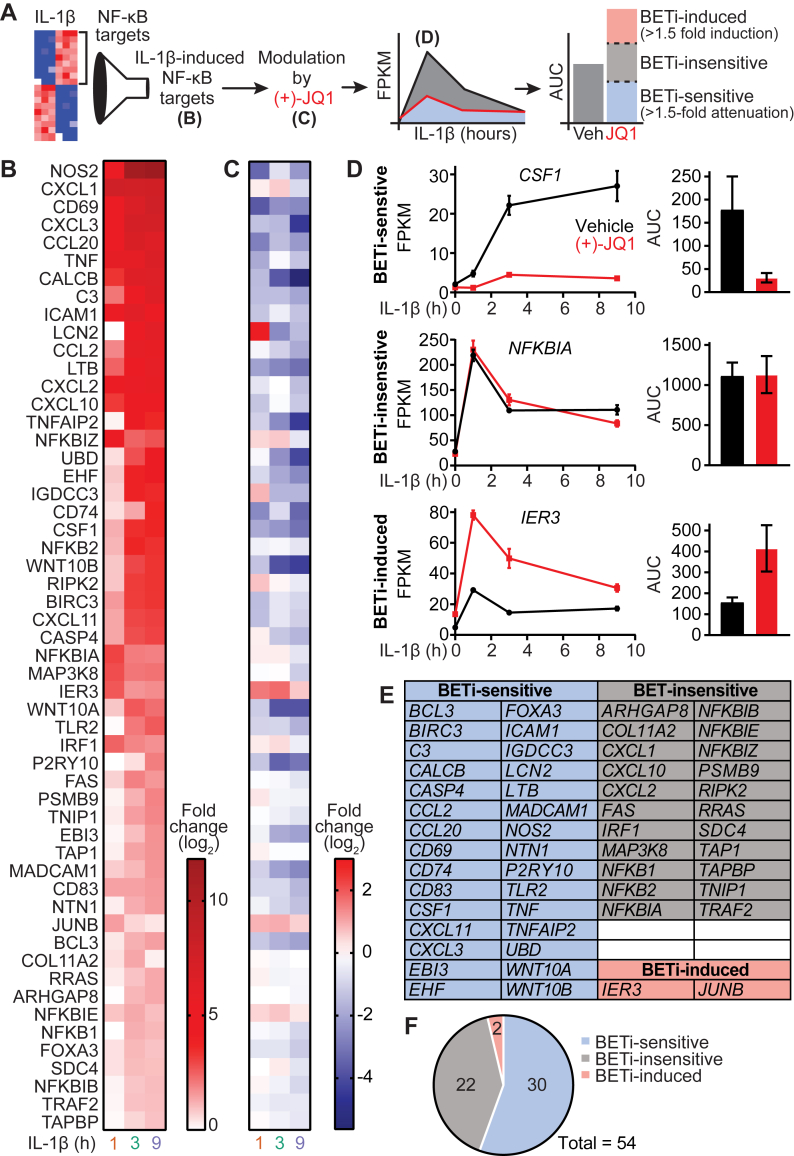
**IL-1-induced NF-κB gene targets display variable attenuation by BET bromodomain inhibition**. *A*, schematic representation of the analysis pipeline. *B*, heatmap displaying IL-1β-induced (fold-change > 1.5; adjusted *p* < 0.05) NF-κB gene targets at 1, 3, and 9 h of 5 U/ml IL-1β exposure. *C*, heatmap displaying differential gene expression by 1-h pretreatment with 0.5 μM (+)-JQ1 *versus* vehicle control on the IL-1β-induced NF-κB gene targets identified in *B*. *D*, plotted gene expression traces for 0.5 μM (+)-JQ1 and vehicle control using FPKM values from RNA-seq. At each treatment time point, the mean and SEM are plotted. Area under the curve calculations were propagated from the time course graphs for select IL-1β-induced NF-κB gene targets plotted as the mean and SD. *E*, table of NF-κB gene targets classified into BETi-sensitive (>1.5 fold attenuation by (+)-JQ1), BETi-insensitive (<1.5 fold induction or attenuation by (+)-JQ1), and BETi-induced (>1.5 fold induction by (+)-JQ1). *F*, pie chart showing the distribution of all IL-1β-induced NF-κB gene targets into defined categories. AUC, area under the curve.

To further assess BET bromodomain inhibition on the regulation of NF-κB gene targets in β-cells, IL-1-stimulated genes were segregated based on the degree of attenuation by (+)-JQ1 (Fig. 6*A*). A comprehensive list of NF-κB regulated genes was assembled by combining the NF-κB-regulated genes identified by GSEA (containing NF-κB binding sites listed in Fig. 4*D*) with the verified NF-κB target gene list assembled by the Gilmore laboratory (https://www.bu.edu/nf-kb/gene-resources/target-genes/). Using this approach, 54 NF-κB genes induced by at least 1.5-fold (adjusted *p* < 0.05) by IL-1 at one or more time points examined were identified (Fig. 6*B*). We then evaluated the effects of (+)-JQ1 on the expression of these NF-κB target genes relative to vehicle control by differential expression analysis (Fig. 6*C*). This analysis revealed three responses to (+)-JQ1: an IL-1-stimulated group of genes that are sensitive to (+)-JQ1, a group of genes insensitive to (+)-JQ1 and a group of genes further induced by (+)-JQ1. The area under the curve was calculated to assess the time-related actions of (+)-JQ1. Three representative genes are shown: *CSF1,* which is sensitive (>1.5-fold repression); *NFKBIA,* which is insensitive; and *IER3,* which is further induced (>1.5-fold induction) by (+)-JQ1 (Fig. 6*A*, schematic of assessment, and *D*). Approximately 56% of IL-1-stimulated NF-κB target genes were attenuated by (+)-JQ1 (BETi-sensitive), ∼41% were unchanged by (+)-JQ1 (BETi-insensitive), and ∼4% were further induced by (+)-JQ1 (BETi-induced) (Fig. 6*F*). These findings indicate that (+)-JQ1 regulates the expression of only a subset of gene targets containing NF-κB consensus sequences. Although the largest group of similarly regulated genes induced by IL-1 contains NF-κB binding motifs, numerous genes, particularly at the later 3- or 9-h treatment time points, are not regulated by NF-κB (Fig. 4*E*, *gray* *bars*). Therefore, the induction and differential expression levels by (+)-JQ1 of these genes were assessed (Fig. S2). (+)-JQ1 also attenuated many of these IL-1-induced, non-NF-κB targets, indicating that BET proteins are also important in the transcription of non-NF-κB targets.

**Figure 7:**
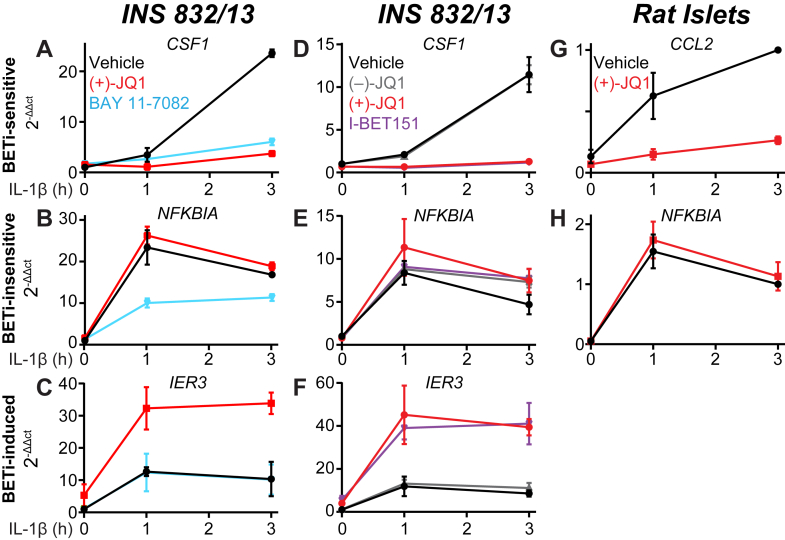
**Comparative effects of NF-κB inhibitors and structurally distinct BET bromodomain inhibitors.** RT-qPCR of (*A*) BETi-sensitive gene *CSF1*, (*B*) BETi-insensitive gene *NFKBIA*, and (*C*) BETi-induced gene *IER3* in INS 832/13 cells treated for 1 h with 0.5 μM (+)-JQ1, 5 μM BAY11-7082, or vehicle, followed by 5 U/ml IL-1β exposure for 0, 1, or 3 h. RT-qPCR of (*D*) BETi-sensitive gene *CSF1*, (*E*) BETi-insensitive gene *NFKBIA*, and (*F*) BETi-induced gene *IER3* in INS 832/13 cells treated for 1 h with 0.5 μM (+)-JQ1, 0.5 μM (−)-JQ1, 1 μM I-BET151, or vehicle, followed by 5 U/ml IL-1β exposure for 0, 1, or 3 h. RT-qPCR of (*G*) BETi-sensitive gene *CCL2* and (*H*) BETi-insensitive gene *NFKBIA* in primary rat islets treated for 1 h with 5 μM (+)-JQ1, or vehicle, followed by 5 U/ml IL-1β exposure for 0, 1, or 3 h. Three biological replicates are represented at each treatment/time point, and the mean and SEM are plotted.

To further validate that BET bromodomain inhibitors regulate the expression of only a subset of NF-κB targets, the effects of (+)-JQ1 on IL-1-induced NF-κB target genes were assayed by reverse transcription-quantitative polymerase chain reaction (RT-qPCR) and compared to direct inhibition of NF-κB. INS 832/13 cells were pre-treated with (+)-JQ1, BAY 11-7082, or vehicle for 1 h, IL-1 was added for 1 or 3 h, and gene expression levels were assessed. The expression of several BETi-sensitive (Fig. 7*A*, *CSF1*; Fig. S3*A*; *NOS2*), BETi-insensitive (Fig. 7*B*, *NFKBIA*; Fig. S3*B*; *IRF1*), and BETi-induced (Fig. 7*C*, *IER3*) genes are shown. For the BETi-sensitive genes, both (+)-JQ1 and BAY 11-7082 similarly attenuated IL-1-induced transcription (Figs. 7*A*/S3*A*). For the BETi-insensitive genes, BAY 11-7082 attenuated transcriptional induction, whereas (+)-JQ1 had no effect (Figs. 7*B*/S3*B*). This confirms that NF-κB regulates genes from these categories as BAY 11-7082 universally attenuates their transcriptional induction, whereas the effects of (+)-JQ1 are gene dependent.

To provide additional support that the observed effects of (+)-JQ1 are through BET bromodomain inhibition, INS 832/13 cells were pre-treated with (+)-JQ1, the inactive enantiomer (−)-JQ1, a structurally distinct BET bromodomain inhibitor I-BET151, or vehicle for 1 h before IL-1 was added for 1 or 3 h. RT-qPCR was used to assess gene expression levels. As anticipated for on-target effects, the expression of BETi-sensitive (Fig. 7*D*, *CSF1*), BETi-insensitive (Fig. 7*E*, *NFKBIA*; Fig. S3*C*, *IRF1*), and BETi-induced (Fig. 7*F*; *IER3*) genes by (+)-JQ1 was recapitulated by I-BET151, and the inactive (−)-JQ1 enantiomer matched the vehicle control. To extend our results in INS 832/13 cells, the effects of (+)-JQ1 and IL-1 were assessed in primary rat islets. Isolated rat islets were pre-treated with (+)-JQ1 or vehicle, IL-1 was added for 1 or 3 h, and RT-qPCR was used to assess gene expression. Importantly, the effects of (+)-JQ1 treatment on several BETi-sensitive (Fig, 7*G*, *CCL2;* Fig. S3*D*; *NOS2*) and BETi-insensitive (Fig. 7*H*, *NFKBIA*; Fig. S3*D*, *IRF1*) genes in primary islets matched those found in INS 832/13 cells.

As several IL-1-induced gene sets attenuated by (+)-JQ1 were associated with exposure to other cytokines (*e.g.* TNFα signaling and interferon responses), we probed the effects of BET bromodomain inhibition on TNFα-stimulation. INS 832/13 cells were pretreated with (+)-JQ1 or vehicle control before adding TNFα for 1 or 3 h. The expression of BETi-sensitive (Fig. S4*A*, *CSF1*), BETi-insensitive (Fig. S4*B*/C, *NFKBIA* and *IRF1*), and BETi-induced (Fig. S4*D*, *IER3*) was assessed by RT-qPCR. The differential attenuation by BET bromodomain inhibition was maintained, suggesting effects of BET bromodomain inhibitors among these targets are not stimulus dependent.

### BET bromodomain inhibitors preferentially attenuate the expression of NF-**κ**B inflammatory gene targets

**Figure 8:**
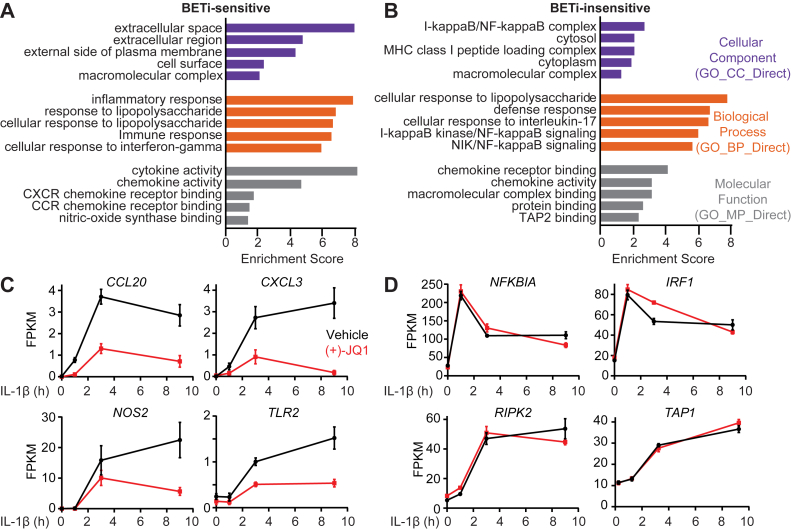
**BET bromodomain inhibitors preferentially attenuate NF-κB targets with defined cellular roles**. *A*, BETi-sensitive genes (from Fig. 6*E*) were subjected to functional annotation clustering using DAVID. The top five enriched GO terms for cellular component (CC), biological process (BP), and molecular function (MF) are shown. *B*, BETi-insensitive genes (from Fig. 6*E*) were subjected to functional annotation clustering using DAVID. The top five enriched GO terms for cellular component (CC), biological process (BP), and molecular function (MF) are shown. *C–F*, plotted gene expression traces for 0.5 μM (+)-JQ1 and vehicle control using FPKM values from select BETi-sensitive NF-κB gene targets. *G–H*, plotted gene expression traces for 0.5 μM (+)-JQ1 and vehicle control using FPKM values from select BETi-insensitive NF-κB gene targets. The mean and SEM are plotted at each treatment time point for line graphs.

GO analysis with DAVID was used to identify the categories of IL-1-induced NF-κB-dependent target genes whose expression is differentially regulated (identified in Fig. 6*E*) by BET bromodomain inhibitors (BETi-sensitive and -insensitive). The top 5 GO terms (ranked by enrichment score) within cellular components (CC), biological processes (BP), and molecular function (MF) categories were plotted and included most genes within both lists (Fig. 8, *A*/B and Table S1/2). This analysis of the BETi-sensitive and -insensitive gene lists was restricted because both lists consist entirely of NF-κB target genes. As anticipated, there were some overlapping GO terms across our gene lists, particularly for larger, comprehensive gene sets with GO terms broadly relating to cellular stress, where NF-κB activation is expected [*e.g.* response to lipopolysaccharide (BP), and chemokine activity (MF)]. However, there were stark differences, particularly in the CC and BP GO terms. IL-1-induced NF-κB genes sensitive to BET bromodomain inhibitors with the highest enrichment scores for CC include extracellular space and region, external side of the plasma membrane, and cell surface. Genes with the highest enrichment scores for BP include inflammatory and immune responses and responses to lipopolysaccharide (LPS) and interferon-γ. Genes with the highest enrichment scores for MF include cytokine and chemokine activity. These findings indicate that (+)-JQ1 attenuates IL-1 stimulated pathways involving extracellular signaling and inflammatory processes. Many of the BETi-sensitive genes participate in autoimmune and inflammatory pathologies like chemokines and chemokine receptors (*CCL20* and *CXCL3*; Fig. 8*C*, Table S1), inflammatory mediators (*NOS2*; Fig. 8*C*, Table S1), and cell surface proteins (*TLR2*, Fig. 8*C*, Table S1).

In contrast, IL-1-stimulated genes insensitive to BET bromodomain inhibitors include pathways responsible for maintaining cellular homeostasis. Overall, the enrichment scores for the CC GO terms are lower but significant and include IκB/NF-κB complex, cytosol/cytoplasm, and MHC class I loading and complex formation (Fig. 8*B*). Enrichment scores for canonical and non-canonical NF-κB signaling, responses to LPS, and defense response are elevated in the BP GO terms, while chemokine receptor and activity are elevated in the MF GO term (Fig. 8*B*). The temporal expression levels of four genes insensitive to (+)-JQ1 involved in NF-κB signaling are shown in Figure 8*D*, and the complete list of genes is provided in Table S2.

The overall trend for the function of IL-1-stimulated genes in INS 832/13 cells sensitive to (+)-JQ1 includes inflammation and immune signaling. IL-1 stimulated genes whose products maintain cellular homeostasis *via* intracellular signaling (*e.g.* IκB, which turns off NF-κB signaling), cytoplasmic and membrane homeostasis, and cellular defense are not inhibited by (+)-JQ1. These findings suggest that BET bromodomain proteins play a significant role in regulating the inflammatory responses stimulated by IL-1 in β-cells. In contrast, genes whose products regulate cellular homeostasis and cellular defense are insensitive to BET bromodomain inhibition.

## Discussion

BET bromodomain inhibitors attenuate the progression of inflammatory diseases in several models (11, 12, 50, 51, 52, 53, 54) and are thought to exert these anti-inflammatory effects by disrupting BET protein-mediated activation of the pro-inflammatory transcriptional factor NF-κB (3, 17, 18, 25). While BET bromodomain inhibitors attenuate NF-κB-dependent gene expression, several NF-κB controlled genes are insensitive to these inhibitors (17, 18, 25). Paradoxically, NF-κB activates inflammatory genes while also controlling the expression of genes governing cell survival, immune function, and anti-inflammatory responses [reviewed in (26)]. Overall, previous studies support selectivity in the mechanisms by which BET proteins regulate NF-κB-dependent transcriptional activation.

Using a well-characterized system in which NF-κB-dependent expression of inflammatory genes and genes known to maintain cellular homeostasis are known, we evaluated whether regulation by BET bromodomain inhibitors is selective for individual classes or types of genes. In particular, we examined the effects of the pan-BET bromodomain inhibitor (+)-JQ1 on IL-1 stimulated gene expression in β-cell-like INS 832/13 cells. Previous studies confirmed that INS 832/13 cells respond to cytokines and BET bromodomain inhibitors in a manner consistent with primary β-cells (55, 56, 57).

In autoimmune diabetes, cytokine-induced NF-κB activation induces inflammatory gene transcription and is implicated in disease onset [reviewed in (58)]. Accordingly, we identified 196 genes that were rapidly induced by IL-1 during the treatment time course (1–9 h) in INS 832/13 cells (Fig. 1), many of which propagate inflammation (Fig. 2). Using GSEA, the most common regulatory element shared among the IL-1-induced genes was *cis-*binding elements for NF-κB (Fig. 4). These findings align with the established role of NF-κB in regulating IL-1-stimulated gene expression [reviewed in (58)] and with our earlier study demonstrating BET proteins as potential transcriptional co-activators of NF-κB target genes (25). To investigate the effects of (+)-JQ1 on these genes, we isolated the 54 IL-1-induced NF-κB target genes and assessed the differential impact of (+)-JQ1 on their expression. Remarkably, approximately 60% of IL-1-induced NF-κB target gene expression was attenuated by (+)-JQ1, while 40% of these genes showed minimal change (<1.5-fold) in expression (Fig. 6*F*). These findings suggest a selective regulatory mechanism through which BET proteins modulate IL-1-stimulated NF-κB-dependent gene expression in β-cells.

DAVID was used to identify the pathways and function (GO terms) of the IL-1-stimulated NF-κB-dependent genes sensitive to BET bromodomain inhibition. These genes are implicated in extracellular signaling and contribute to inflammatory and immune responses (Fig. 8*A* and Table S1). Many IL-1-induced genes sensitive to BET bromodomain inhibition have well-defined roles in autoimmune and inflammatory disease progression. These include the inflammatory mediator *NOS2* that directs the production and accumulation of potentially damaging levels of nitric oxide (59), chemokines *CCL20* and *CXCL3* that recruit inflammatory immune cells (60, 61, 62), and the cell surface protein *TLR2* that recognizes bacterial products, including peptidoglycan and lipoproteins (Fig. 8*C*). Several genes coding for chemokines, including *CXCL2* and *CXCL10,* were not attenuated by BET bromodomain inhibitors using the 1.5-fold area under the curve cutoff. Enhanced levels of these chemokines are associated with developing autoimmune diabetes by recruiting and activating immune cells (63, 64). While BET bromodomain inhibition did not achieve the 1.5-fold threshold, we did observe a decrease in expression of 1.4-fold for *CXCL2* and 1.3-fold for *CXCL10* in response to BET bromodomain inhibition (Table S3).

Of the approximately 40% of IL-1-stimulated NF-κB dependent genes not sensitive to BET protein inhibition, the GO analysis indicated these genes participate in the control of intracellular homeostasis, including NF-κB signaling (IκB), cellular defense, and recovery from oxidative damage (Fig. 8*B* and Table S2). Specific genes include four family members of IκB (inhibitor of NF-κB) that block nuclear NF-κB translocation to ensure the transitory nature of NF-κB-driven transcription. We also identified genes involved in transcriptional activation (*IRF1*) and signal transduction (*RIPK2*). IRF1 is interesting not only because IL-1 induces its expression but also because of its primary role in controlling antiviral responses (65). A protective role for IRF1 in autoimmune diabetes is supported by findings that when IRF1 is expressed, it attenuates islet inflammation, enhances the expression of genes that protect β-cells from environmental stressors, and facilitates the maintenance of β-cell identity (66, 67, 68). Taken together, our RNA-sequencing studies indicate that subsets of NF-κB target genes with inflammatory *versus* homeostatic roles use different regulatory strategies for initiating transcription.

NF-κB transcriptional subunits that influence gene selectivity and transcriptional output. Such modifications may define the disparate requirements of BET proteins at select targets. One of the most well-studied is the acetylation status of the p65 subunit of NF-κB, where seven lysine acetylation sites have been identified with variable influences on transcriptional activity and promoter specificity [reviewed in (69)]. BRD4 can interact with acetylated p65 (17, 18). Different combinations of acetylated residues may initiate the transcription of subsets of NF-κB targets by different mechanisms, with variable dependencies of BET bromodomain inhibitors. Future studies addressing these possibilities will further elucidate the mechanisms of transcriptional initiation among subsets of NF-κB target genes and the cofactors required.

This study has several limitations. First, RNA-seq analyses were performed exclusively in the INS 832/13 cell line and with a single BET bromodomain inhibitor, (+)-JQ1. To circumvent these limitations, gene expression was validated by RT-qPCR for critical genes contained within our subsets of differentially BETi-sensitive genes list with a structurally distinct BET inhibitor, I-BET151 (Fig. 7, *D*–*F*) and in primary rat islets (Fig. 7, *G*/H). Another limitation is our relatively narrow focus on NF-κB target genes. Although many of the deleterious effects of IL-1 are linked to NF-κB-mediated transcription (29, 30, 31), and the protective effects of BET bromodomain inhibitors are linked to attenuation of those genes (3, 16, 17, 18), there may be alternative effects of BET bromodomain inhibitors relating to β-cell health. Indeed, we identified non-NF-κB-regulated genes modulated by (+)-JQ1, including genes induced by IL-1 (Fig. S2). We also identified gene sets relating to cellular metabolism unaffected by IL-1 but induced by BET bromodomain inhibitors (Fig. 3*H*). Given that some of these non-NF-κB genes also have roles in the inflammatory progression and metabolic capacity of the β-cell, further studies of how BET proteins regulate these targets are warranted.

In summary, this study demonstrates that cytokine-stimulated NF-κB target genes in β-cells differentially depend on BET proteins for maximal transcriptional activation. The unique cellular role informs these disparate dependencies of each gene. Genes mediating extracellular and inflammatory responses require BET proteins for full transcriptional activation. In contrast, the transcription of genes that maintain the integrity of the intracellular process, including the NF-κB signaling pathway, and genes that play protective roles or may maintain β-cell identity are independent of BET proteins. Continuing to elucidate the mechanisms by which BET proteins differentially regulate the expression of NF-κB target gene subclasses in β-cells will advance the application of more BET bromodomain inhibitors in the transcriptional regulation of autoimmune diabetes and other inflammation-driven diseases.

## Experimental procedures

### Cell culture

INS 832/13 rat insulinoma cells were cultured in RPMI 1640 supplemented with 10% v/v fetal bovine serum (FBS, Gemini Bio), 2 mM L-glutamine, 1 mM sodium pyruvate, 10 mM HEPES, 50 μM 2-mercaptoethanol, 100 units/ml penicillin, and 100 μg streptomycin. Cells were grown in an atmosphere of 37 °C and 5% CO_2_. At confluency (∼70–80%), cells were lifted using 0.05% w/v trypsin and plated at 500,000 cells/ml for experimental treatments unless otherwise noted. Cells were routinely tested using the MycoAlert PLUS Kit (Lonza) to ensure all data was obtained from mycoplasma-free cells.

### RNA-sequencing

INS 832/13 rat insulinoma cells were subjected to 1-h pretreatment with 500 nM (+)-JQ1 or DMSO (vehicle control), followed by 5 U/ml IL-1β for 0, 1, 3, or 9 h. Total RNA was isolated *via* the RNeasy mini kit (Qiagen). RNA was collected from three independent experiments. RNA quality was validated using an RNA ScreenTape on a TapeStation (Agilent Technologies). RNA concentration was determined by NanoDrop, and 1 μg of total RNA was used for library preparation with ERCC RNA (ThermoFisher Scientific) added to each sample before mRNA isolation using the NEBNext Poly(A) mRNA Magnetic Isolation Module (New England Biolabs). Libraries were prepared according to the manufacturer's specifications using the NEBNext Ultra RNA Library Prep Kit for Illumina (New England Biolabs) and NEBNext Multiplex Oligos for Illumina (New England Biolabs). The average library length was determined using a DNA ScreenTape on a TapeStation. Library concentration was determined by the NEBNext Library Quant Kit for Illumina (New England Biolabs). Paired-end sequencing (76 cycles) on libraries was performed on a NextSeq 500 (Illumina). Raw sequences (FASTQ files) were aligned to the rat reference genome rn7.2 using STAR (70), differential expression was performed using DE-seq (71), and gene-level absolute expression was quantified using CuffLinks (72). Analysis was done using default parameters through Basepair (www.basepairtech.com).

### Gene set enrichment analysis

Gene Set Enrichment Analysis (GSEA) was performed on our RNA-seq data using the Broad Institute's GSEA software v4.3.3 (35, 36) and MsigDB gene set collections (73, 74) to define significantly enriched gene sets. Hallmark gene sets (h.all.v2023.2) and Regulatory target gene sets (c3.all.v2023.2) were used. Rat Ensembl Gene IDs were mapped to human gene symbols, as the MsigDb and associated gene sets are based on human gene annotations. GSEA ranks all genes within a sample dataset based on the differential expression between the two experimental groups to give enrichment scores (ES) and normalized enrichment scores (NES), which consider differences in pathway size, allowing for comparison across pathways. As recommended by the Broad Institute, this analysis involved 1000 gene set permutations with gene sets limited to 15 to 500. A nominal *p*-value of 0.01 and a FDR of 10% were used for all bioinformatic analyses.

### Functional annotation clustering analysis

Lists of IL-1-induced (Fig. S1) or BETi-sensitive and -insensitive (Fig. 6*E*) gene lists were input into the Database for Annotation, Visualization, and Integrated Discovery (DAVID) web interface and submitted for functional annotation using default settings. Direct GO annotations were used and given an enrichment score defined as the geometric mean (in -log scale) of the *p*-value. For the IL-1-induced gene list (Fig. S1), the top 10 direct GO terms were plotted. For the BETi-sensitive and -insensitive gene lists (Fig. 6*E*), the top five enriched GO categories, cellular component (CC_Direct), biological process (BP_Direct), and molecular function (MF_Direct) were plotted for each gene list.

### RNA isolation and real-time quantitative polymerase chain reaction analysis

Total RNA was isolated from INS 832/13 cells after indicated treatment conditions using the RNeasy mini kit (Qiagen). Isolated RNA samples were treated with DNase using a TURBO DNA-free kit (Invitrogen). Complementary DNA (cDNA) was synthesized using Maxima H Minus Reverse Transcriptase (Thermo Fisher Scientific, Waltham, MA). Using these cDNA as templates, RT-qPCR analysis was completed using the primers listed in Table S4 (Integrated DNA Technologies) and EvaGreen qPCR master mix (Midwest Scientific) and run on a CFX96 Real-Time System (Bio-Rad) with the following cycle conditions: 95 °C for 3 min, then 40 cycles of 95 °C for 15 s, 64 °C for 30 s, and 72 °C for 35 s. Each gene was assayed in 3 to 5 independent biological replicates with GAPDH for normalization. Data is normalized to an internal reference condition for fold change (2^−ΔΔCT^).

### Chemical inhibition of BET bromodomains, NF-**κ**B, IL-1**β**, and TNF**α** treatments

BET bromodomain inhibitors (+)-JQ1 (eNovation) and I-BET151 (a gift from GlaxoSmithKline) and NF-κB inhibitor BAY 11-7082 (Millipore Sigma) were dissolved in DMSO. An equal volume of DMSO or the inactive enantiomer (−)-JQ1 (eNovation) was used as a control. All treatments were completed in ≤0.5% v/v final DMSO concentration in the corresponding media. A concentration of 0.5 μM (+)-JQ1 was used for cell culture treatments based on previously published concentration-response experiments (46, 75). A concentration of 1 μM of I-BET151 was used for cell culture treatments, as our collaborators at GlaxoSmithKline recommended (13, 76, 77). A 5 μM BAY 11-7082 concentration was used based on previous DNA-binding experiments (78). Recombinant IL-1β (Peprotech, Cranbury, NJ) was reconstituted in RPMI at 0.1 μg/ml, which we determined was the equivalent of 1000 units/ml (U/ml) *via* in-house Griess assay. All IL-1β treatments were conducted at 5 U/ml. Recombinant TNFα (Peprotech, Cranbury, NJ) was reconstituted at 10 μg/ml, with treatments conducted at 50 ng/ml.

### Luciferase reporter assays

INS 832/13 cells were transfected with 0.3 μg of p1242 3x-KB-L plasmid (a gift from Bill Sugden; Addgene plasmid #26699; https://n2t.net/addgene:26,699; RRID: Addgene_26699) (57) per 500,000 cells using Avalanche-Omni transfection reagent (EZ-Biosystems). Forty-eight hours after transfection, BET bromodomain or NF-κB inhibitors were added for 1 h before adding IL-1β (5 U/ml) for 5 h. Luciferase activity was determined *via* the Luciferase Assay System (Promega Corporation) and read on a BioTek Cytation 5 multi-mode microplate reader using a Luminescence filter cube (BioTek, Winooski, VT). DMSO vehicle (≤0.5% v/v) with 5 U/ml IL-1β was used as an internal control, and the signal was set equal to 100%.

### Nuclear extraction and gel shift analysis

After treatment of INS 832/13 cells with IL-1β (5 U/ml) for 30 min, nuclear proteins were extracted as previously described (79). IRDye 700 NF-κB consensus oligonucleotides (5′-AGTTGAGGGGACTTTCCCAGGC-3′; 3′-TCAACTCCCCTGAAAGGGTCCG-5′) (LI-COR, Lincoln, NE) were used to probe binding. Binding reactions used 4 μg nuclear lysate, 50 fmol oligonucleotide. p65 (8242, Cell Signaling) or two distinct BRD4 (1, A300–985, Bethyl Laboratories, Montgomery, TX or 2, AB128874. Abcam, Cambridge, MA) antibodies were preincubated with nuclear lysate for 20 min at 4 °C before the addition of DNA probe for an additional 20 min. Binding reactions were separated on a 4.5% native polyacrylamide gel containing 50 mM Tris (pH 7.5), 0.38 M glycine, and 2 mM EDTA in a 0.5% TBE buffer system (Bio-Rad) before visualizing on an Odyssey Fc imager and Image Studio ver. 5.2 software (LI-COR).

### Isolation and culture of primary rat islets

Pancreatic islets were harvested from male Sprague Dawley rats (Charles River) *via* collagenase digestion as previously described (80). Isolated islets were cultured in CMRL-1066 (ThermoFisher) supplemented with 10% v/v FBS, 2 mM L-glutamine, 100 units/ml penicillin, and 100 μg streptomycin. For RT-qPCR experiments, 25 to 50 islets were used per treatment condition in 300 μl CMRL-1066. All studies were approved by the Institutional Animal Care and Use Committee by the ethical review process at the Medical College of Wisconsin, where the work was performed.

## Data availability

All sequencing data have been deposited in the Gene Expression Omnibus database (GEO) and are publicly available under the series GSE286117.

## Supporting information

This article contains supporting information.

## Conflict of interest

The authors declare that they have no conflicts of interest with the contents of this article.
