# Supplementary material for: BET bromodomain inhibitors attenuate transcription of a subset of IL-1-induced NF-κB targets that promote inflammation in β-cells
Source: J Biol Chem. 2025 Jun 10;301(7):110358. doi: 10.1016/j.jbc.2025.110358 (PMC12270673; doi:10.1016/j.jbc.2025.110358)
Supplement: Supporting information [file mmc1.pdf]

Supporting Information

BET bromodomain inhibitors attenuate transcription of a subset of IL-1-induced NF-κB targets that promote inflammation in β-cells

Joshua A. Nord[1], Savannah J. Makowski[1], Paul F. W. Sidlowski[1], Karina L Bursch[1], John A. Corbett[1], and Brian C. Smith[1,2,3*]

[1]Department of Biochemistry, Medical College of Wisconsin, Milwaukee, WI 53226, USA;

[2]Structural Genomics Unit, Linda T. and John A. Mellowes Center for Genomic Sciences and Precision Medicine, Medical College of Wisconsin, Milwaukee, WI 53226; [3]Program in Chemical Biology, Medical College of Wisconsin, Milwaukee, WI 53226

***Corresponding author:** Brian C. Smith, Email: brismith@mcw.edu

Supplemental Figures and Tables

Figure S1. DAVID analysis of GO terms enriched by IL-1 in INS 832/13 cells

Figure S2. IL-1-induced non-NF-κB genes and modulation by (+)-JQ1 in INS 832/13 cells.

Figure S3. RT-qPCR analysis of IL-1-induced NF-κB gene targets.

Figure S4. Analysis of TNFα-induced NF-κB gene targets in INS 832/13 cells.

Table S1. BETi-sensitive IL-1-induced NF-κB genes within enriched GO categories by GSEA.

Table S2. BETi-insensitive IL-1-induced NF-κB genes within enriched GSEAGO categories by GSEA.

Table S3. (+)-JQ1-induced fold modulation of IL-1-induced NF-κB gene targets.

Table S4. Primer sequences used for RT-qPCR experiments.

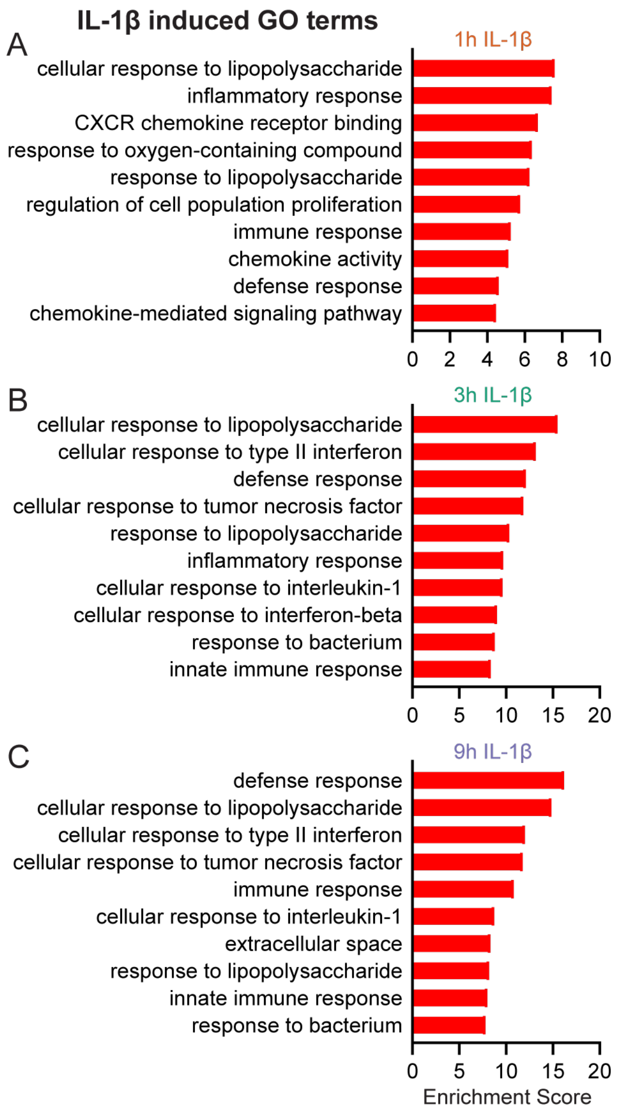

Figure S1. DAVID analysis of GO terms enriched by IL-1 in INS 832/13 cells. After indicated exposure to 5 U/mL IL-1β (**A**, 1 hour; **B**, 3 hours; **C**, 9 hours), significantly enriched genes were subjected to gene ontology analysis using DAVID. The top 10 terms included for each treatment time point.

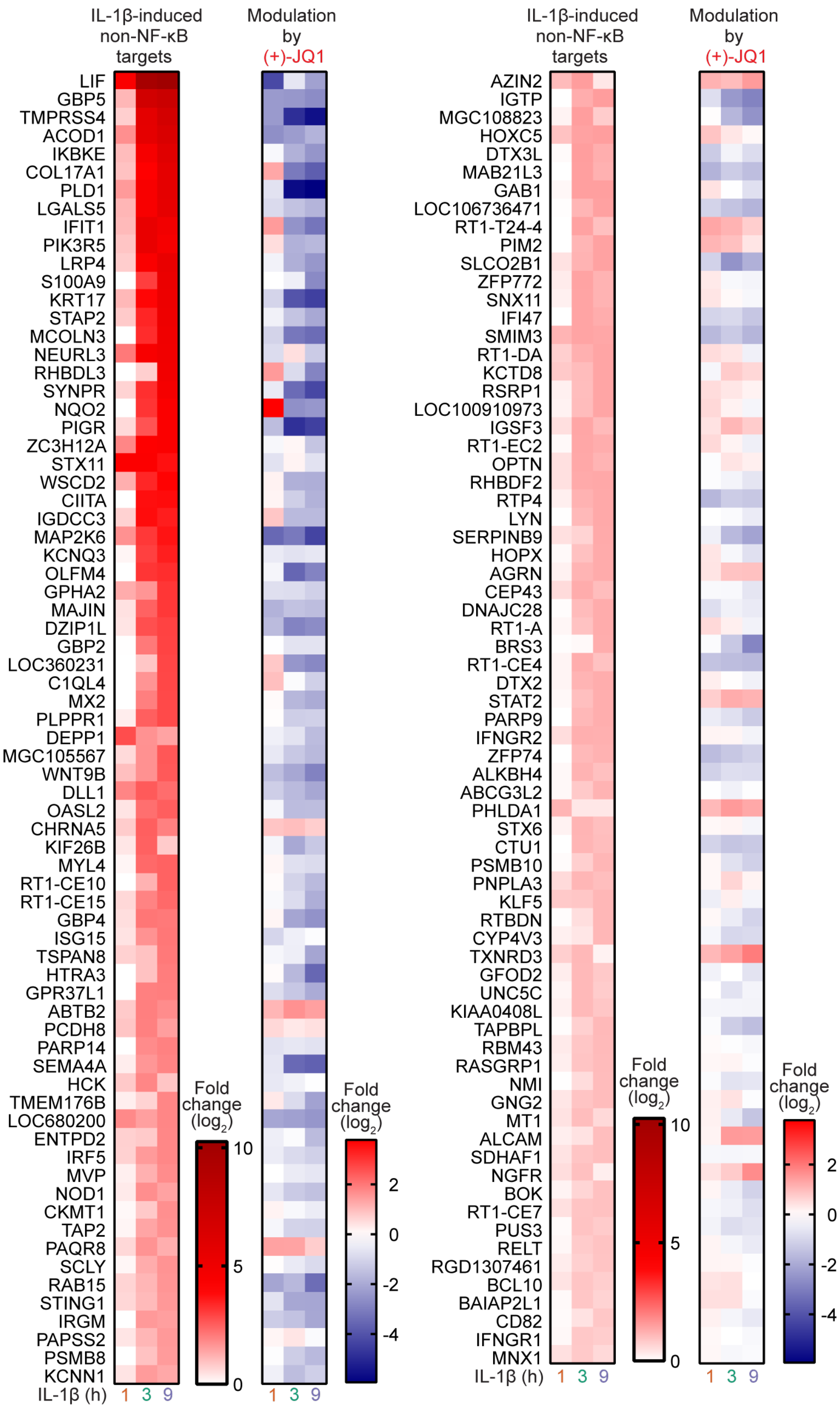

Figure S2. IL-1-induced non-NF-κB genes and modulation by (+)-JQ1 in INS 832/13 cells. Heatmaps display IL-1β-induced (fold-change ≥1.5; adjusted $p < 0.05$) non-NF-κB gene targets at 1, 3, and 9 hours of 5 U/mL IL-1β exposure or differential gene expression by 1-hour pretreatment with 0.5 μM (+)-JQ1 vs vehicle control on the IL-1β-induced NF-κB gene targets.

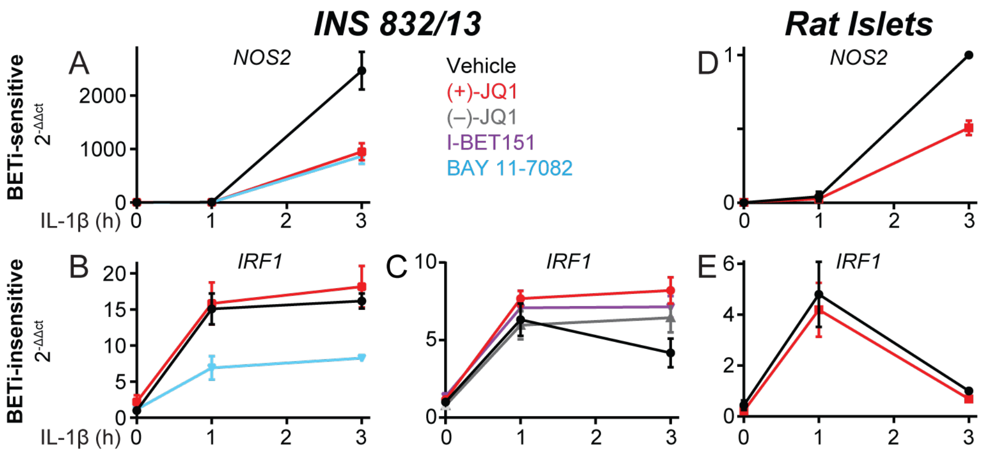

Figure S3. RT-qPCR analyses of IL-1-induced NF-κB gene targets in INS 832/13 cells. RT-qPCR of **(A)** BETi-sensitive gene *NOS2* and **(B)** BETi-insensitive gene *IRF1* in INS 832/13 cells treated for 1 hour with 0.5 μM (+)-JQ1, 5 μM BAY11-7082, or vehicle, followed by 5 U/mL IL-1β exposure for 0, 1, or 3 hours. RT-qPCR of **(C)** BETi-insensitive gene *IRF1* in INS 832/13 cells treated for 1 hour with 0.5 μM (+)-JQ1, 0.5 μM (–)-JQ1, 1 μM I-BET151, or vehicle, followed by 5 U/mL IL-1β exposure for 0, 1, or 3 hours. RT-qPCR of **(D)** BETi-sensitive gene *NOS2* and **(E)** BETi-insensitive gene *IRF1* in primary rat islets treated for 1 hour with 5 μM (+)-JQ1, or vehicle, followed by 5 U/mL IL-1β exposure for 0, 1, or 3 hours. Three biological replicates are represented at each treatment/time point, and the mean and SEM are plotted.

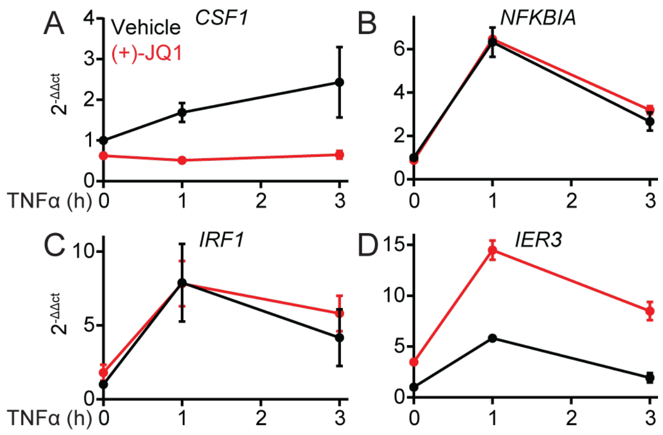

Figure S4. Analysis of TNFα-induced NF-κB gene targets in INS832/13 cells. RT-qPCR of **(A)** BETi-sensitive gene *CSF1*, **(B/C)** BETi-insensitive genes *NFKBIA and IRF1*, and **(D)** BETi-induced gene *IER3* in INS 832/13 cells treated for 1 hour with 0.5 μM (+)-JQ1, or vehicle, followed by 5 U/mL IL-1β exposure for 0, 1, or 3 hours.

Table S1. BETi-sensitive IL-1-induced NF-κB genes within enriched GO categories by GSEA.

Cellular Component (GO_CC_Direct)		
GO Term (Direct)	P-Value	Gene Name
Extracellular Space	9.81E-09	*CCL2*
		CCL20
		CXCL11
		CXCL3
		CD74
		WNT10A
		WNT10B
		CALCB
		CSF1
		C3
		ICAM1
		LCN2
		LTB
		NOS2
		TNF
Extracellular Region	1.61E-05	*CCL2*
		EBI3
		WNT10A
		WNT10B
		CALCB
		CSF1
		C3
		LCN2
		NTN1
		TNF
External side of plasma membrane	4.85E-05	*CD74*
		CD83
		CD69
		EBI3
		ICAM1
		TLR2
		TNF
Cell surface	3.66E-03	*CD74*
		CD83
		C3
		ICAM1
		TLR2
		TNF
Macromolecular complex	8.04E-03	*BCL3*
		CD74
		CD69
		BIRC3
		CASP4
		C3

Biological Process (GO_BP_Direct)		
GO Term (Direct)	P-Value	Gene Name
Inflammatory response	1.42E-08	*CCL2*
		CCL20
		CXCL11
		CXCL3
		CSF1
		C3
		NOS2
		TLR2
		TNF
Response to lipopolysacchar-ide	1.58E-07	*CCL2*
		CXCL3
		CSF1
		ICAM1
		LCN2
		NOS2
		TLR2
		TNF
Cellular response to lipopolysacchar-ide	2.38E-07	*CCL2*
		CCL20
		CXCL11
		CXCL3
		ICAM1
		LCN2
		NOS2
		TNF
Immune response	2.77E-07	*CCL2*
		CCL20
		CXCL11
		CXCL3
		CD74
		LTB
		TLR2
		TNF
Cellular response to interferon-gamma	1.08E-06	*CCL2*
		CCL20
		ICAM1
		NOS2
		TLR2
		TNF

Molecular Function (GO_MP_Direct)		
GO Term (Direct)	P-Value	Gene Name
Cytokine activity	7.30E-09	*CCL2*
		CCL20
		WNT10A
		WNT10B
		CSF1
		LTB
		NTN1
		TNF
Chemokine activity	2.10E-05	*CCL2*
		CCL20
		CXCL11
		CXCL3
CXCR chemokine	1.74E-02	*CXCL1*
		CXCL3
CCR chemokine receptor binding	3.03E-02	*CCL2*
		CCL20
Nitric-oxide synthase binding	3.74E-02	*CD74*
		NOS2

Table S2. BETi-insensitive IL-1-induced NF-κB genes within enriched GO categories by GSEA.

Cellular Component (GO_CC_Direct)		
GO Term (Direct)	P-Value	Gene Name
I-kappaB/NF-kappaB complex	2.03E-03	*NFKBIA*
		NFKBIB
Cytosol	8.45E-03	*FAS*
		NFKBIA
		NFKBIE
		TNIP1
		TRAF2
		MAP3K8
		NFKB1
		NFKB2
		PSMB9
		RIPK2
MHC class I peptide loading	9.12E-03	*TAPBP*
		TAP1
Cytoplasm	1.44E-02	*NFKBIA*
		NFKBIB
		NFKBIE
		ARGHAP8
		TNIP1
		TRAF2
		IRF1
		MAP3K8
		NFKB1
		NFKB2
		PSMB9
		RIPK2
Macromolecular complex	6.26E-02	*NFKBIA*
		TRAF2
		NFKB1
		RIPK2

Biological Process (GO_BP_Direct)		
GO Term (Direct)	P-Value	Gene Name
Cellular response to lipopolysacchar-ide	1.97E-08	*CXCL1*
		CXCL10
		CXCL2
		NFKBIB
		NFKBIZ
		TNIP1
		NFKB1
		RIPK2
Defense response	2.50E-07	*CXCL1*
		CXCL10
		CXCL2
		TAPBP
		TAP1
Cellular response to interleukin-17	2.97E-07	*CXCL1*
		CXCL10
		NFKBIZ
		NFKB1
I-kappaB kinase/NF-kappaB signaling	1.11E-06	*NFKBIA*
		IRF1
		NFKB1
		NFKB2
		RIPK2
NIK/NF-kappaB signaling	2.68E-06	*NFKBIA*
		TRAF2
		NFKB1
		NFKB2

Molecular Function (GO_MP_Direct)		
GO Term (Direct)	P-Value	Gene Name
Chemokine receptor binding	8.07E-05	*CXCL1*
		CXCL10
		CXCL2
Chemokine activity	8.01E-04	*CXCL1*
		CXCL10
		CXCL2
Macromolecular complex binding	8.20E-04	*FAS*
		NFKBIA
		RRAS
		TRAF2
		NFKB1
		TAP1
Protein binding	2.47E-03	*FAS*
		RRAS
		TRAF2
		IRF1
		NFKB1
		NFKB2
		SDC4
		TAP1
TAP2 binding	4.54E-03	*TAPBP*
		TAP1

Table S3. (+)-JQ1-induced fold modulation of IL-1-induced NF-κB gene targets.

Gene Name	Fold Attenuation/ Induction	
CALCB	-15.5	
WNT10B	-11.3	
WNT10A	-9.79	
UBD	-8.30	
LTB	-7.03	
TNFAIP2	-6.67	
P2RY10	-6.23	
CSF1	-5.71	
CD69	-5.58	
CXCL3	-4.93	
EHF	-4.26	
CD74	-4.15	
MADCAM1	-4.06	
CCL20	-3.26	
BCL3	-3.24	BETi-sensitive
C3	-3.23	
LCN2	-3.09	
EBI3	-3.01	
IGDCC3	-2.73	
CASP4	-2.60	
CCL2	-2.48	
TLR2	-2.32	
NOS2	-2.29	
CD83	-2.06	
NTN1	-1.92	
CXCL11	-1.64	
TNF	-1.64	
FOXA3	-1.64	
BIRC3	-1.59	
ICAM1	-1.51	
CXCL2	-1.37	
CXCL10	-1.35	
TRAF2	-1.34	
NFKB1	-1.33	
NFKBIB	-1.33	
TNIP1	-1.29	
MAP3K8	-1.24	
TAPBP	-1.21	
NFKB2	-1.20	
FAS	-1.17	
PSMB9	-1.10	BETi-insensitive
COL11A2	-1.08	
RRAS	-1.05	
ARHGAP8	-1.03	
SDC4	-1.02	
RIPK2	-1.01	
TAP1	1.01	
NFKBIA	1.02	
CXCL1	1.10	
IRF1	1.13	
NFKBIZ	1.21	
NFKBIE	1.47	
JUNB	1.78	BETi-induced
IER3	2.66	

Table S4. Primer sequences used for RT-qPCR experiments.

Gene target	Forward primer (5′-3′)	Reverse primer (5′-3′)
CCL2	GTATTTGTCACCAAGCTCAAGAG	TGAAGTCCTTAGGGTTGATGC
CSF1	GACAGATGAGAAGGAGCAGAAG	GCTGTCCCACCCTTTGAAT
NOS2	CGAGACTTCTGTGACACACAGC	CATCTCCTGGTGGAACACAGGG
IRF1	AACTCCGTGCCTCATTCC	TCTCTAGCCAGGGTCTCATTC
NFKBIA	GAGGATTACGAGCAGATGGTG	ACCTGACCAATCACTTCCATG
IER3	GATTTTCACCTTCGACCCC	CGAGCAGGAGAAAGAGGATC
GAPDH	GACATCAAGAAGGTGGTGAAGC	TCCAGGGTTTCTTACTCCTTGG
